# Deaths by Chloroform

**Published:** 1853-08

**Authors:** 


From the Southern Medical and Surgical Journal.

*Deaths by Chloroform.*—The New York Journal of Medicine contains a tabular statement of thirty-three cases of death from Chloroform recorded in the European and American periodicals up to the end of 1852, not including "those imperfectly reported, or those in which the persons have taken it without the advice of a professional person." From these tables, it appears that the anæsthetic was administered fatally ten times for operations on the toes and fingers, six times for the extraction of teeth, three times for operating on fistula in ano and hemorrhoids, twice for the application of caustic, once for opening abscess, and eleven times for other operations. Ten of the deaths occurred before the operation was performed.

